# Gastrodia Elata Bl Attenuates Cocaine-Induced Conditioned Place Preference and Convulsion, but not Behavioral Sensitization in Mice: Importance of GABA_A_ Receptors

**DOI:** 10.2174/157015911795017326

**Published:** 2011-03

**Authors:** E.-J Shin, J.-H Bach, T.-T.L Nguyen, B.-D Jung, K.-W Oh, M. J Kim, C.G Jang, S.F Ali, S.K Ko, C.H Yang, H.-C Kim

**Affiliations:** 1 Neuropsychopharmacology and Toxicology Program, College of Pharmacy, Kangwon National University, Chunchon 200-701, Korea; 2 School of Veterinary Medicine, Kangwon National University, Chunchon 200-701, South Korea; 3 College of Pharmacy, Core Research Institute, Chungbuk National University, Cheongju 361-763, South Korea; 4 Division of Bio-Resources Technology, Kangwon National University, Chunchon 200-701, South Korea; 5 Department of Pharmacology, College of Pharmacy, Sungkyunkwan University, Suwon440-746, South Korea; 6 Neurochemistry Laboratory, Division of Neurotoxicology, National Center of Toxicological Research, Food and Drug Administration, Jefferson, AR 72079, USA; 7 Department of Oriental Medical Food & Nutrition, Semyung University, Jecheon 390-711, South Korea; 8 Department of Physiology, College of Oriental Medicine, Daegu Haany University, Daegu 706-828, South Korea

**Keywords:** *Gastrodia elata* Bl, cocaine, seizure, conditioned place preference, GABA_A_ receptors.

## Abstract

It has been suggested that GABAergic neurotransmission can modulate cocaine dependence and seizure activity. Since *Gastrodia elata* Bl (GE), an oriental herb agent, has been shown to enhance GABAergic transmission, we examined whether GE affects cocaine-induced seizures, conditioned place preference (CPP), and behavioral sensitization in mice. Treatment with GE (500 or 1000 mg/kg, p.o.) significantly delayed seizure onset time and significantly shortened seizure duration induced by cocaine (90 mg/kg, i.p.). In addition, cocaine (15 mg/kg, i.p.)-induced CPP was significantly attenuated by GE in a dose-dependent manner. However, GE did not significantly alter behavioral sensitization induced by cocaine (15 mg/kg, i.p.). In order to understand whether GABAergic receptors are implicated in GE-mediated pharmacological action in response to cocaine, GABA_A_ receptor antagonist bicuculline and GABA_B_ receptor antagonist SCH 50911 were employed in the present study. GE-mediated attenuations on the cocaine-induced seizures and CPP were significantly reversed by bicuculline (0.25 or 0.5 mg/kg, i.p.), but not by SCH 50911 (1.5 or 3.0 mg/kg, i.p.). Therefore, our results suggest that GE attenuates cocaine-induced seizures and CPP via, at least in part, GABA_A_ receptor activation.

## INTRODUCTION

Cocaine is a widely used psychostimulant. According to the United Nations Office on Drugs and Crime (UNODC), the total number of people who used cocaine at least once in 2007 is estimated maximally 21 million [1]. It has been suggested that multiple neurotransmitter systems are involved in the cocaine addiction [2] and cocaine-elicited seizures [3], one of the consequences of cocaine intoxication. For instance, extensive findings indicated that cocaine dependence is primarily mediated by enhanced dopaminergic transmission, especially in the mesocorticolimbic pathway [4], but cocaine-induced dopaminergic neurotransmission and behavioral changes can be modulated by GABAergic [5] or glutamatergic [6] innervations to the mesocorticolimbic area. Previous studies also have shown that cocaine-induced seizures can be mediated by enhanced dopaminergic and glutamatergic transmissions [7] and reduced GABAergic transmission [8].

*Gastrodia elata* Blume (GE), a traditional oriental herbal agent, has long been used for epilepsy, stroke, and other neurological disorder in Asian countries, and its major components are gastrodin, p-hydroxybenzylaldehyde, p-hydroxybenzylalcohol, vanillyl alcohol, vanillin, and etc. [9]. A number of *in vitro* and *in vivo* studies have suggested therapeutic potentials of GE and its individual components against epileptic seizure/convulsion [10], cerebral ischemia [11], anxiety [12], and depression [13]. GE or its active components could exert pharmacological effects via anti-oxidation [14], anti-inflammation [15], and modulation of monoaminergic [13] and amino acid [16] neurotransmitter systems. Especially, GE has been shown to increase the extracellular GABA levels in *in vivo* microdialysis study, and to consequently enhance the GABAergic neurotransmission [17]. Earlier study suggested that activation of GABA_A_ receptor is important for GE-mediated anxiolytic effect in mice [12].

Since GABA-mimetic drugs and GABAergic agonists have shown to attenuate behavioral sensitization or seizures induced by cocaine [18, 19], we examined whether GE affects cocaine-induced seizures, CPP, and behavioral sensitization in mice. In addition, we examined whether GABAergic receptors are involved in GE-mediated pharmacological action in response to cocaine.

## METHODS

All animals were treated in accordance with the NIH *Guide for the care and use of laboratory animals* (NIH Publication No. 85-23, 1985; www.dels.nas.edu/ila). This study was performed in accordance with the Institute for Laboratory Animal Research (ILAR) guidelines for the care and use of laboratory animals. Male C57BL/6J mice (Bio Genomic Inc., Charles River Technology, Gapyung-Gun, Gyeonggi-Do, South Korea) weighing 25 ± 3 g were maintained on a 12 h:12 h light:dark cycle and fed *ad libitum*.

Cocaine hydrochloride (Hansaem Pharmaceutical Company, Seoul, South Korea) and SCH 50911 (Tocris bioscience, Ellisville, MO, USA) were dissolved in 0.9 % sterile saline. Methanol extract of GE was obtained from Samsung Herb Medicine, Co. (Chunchon, South Korea) and suspended in 0.5 % carboxymethylcellulose. (+)-Bicuculline (Tocris bioscience, Ellisville, MO, USA) was dissolved in saline acidified to pH 3 using 0.1 N HCl. All solutions were immediately prepared before use. Experimental schedules are shown in Fig. (**1**).

CPP and behavioral sensitization were performed as described previously [20]. An automated video-tracking system (Noldus Information Technology, Wageningen, The Netherlands) was employed to record and analyze the movements of mice.

Statistical analyses were performed using one-way analysis of variance (ANOVA) or *Chi *square test. A *post-hoc* Fisher’s PLSD test was followed. A *P*-value <0.05 was accepted as statistically significant.

## RESULTS AND DISCUSSION

### GE Attenuated Cocaine-Induced Seizures

**Fig. (2):**
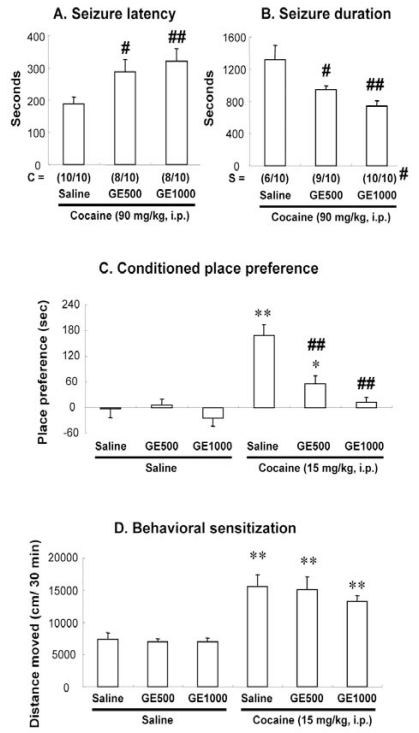
Effect of GE on the cocaine-induced seizure latency (**A**), seizure duration (**B**), CPP (**C**), and behavioral sensitization (**D**). GE 500 or GE 1000 = GE 500 or 1000 mg/kg, C = convulsing ratio, S = survival ratio. Each value is the mean ± S.E.M. of 10 (A and B) or 12 mice (C and D). ^*^P < 0.05, ^**^P < 0.01 vs. Saline + Saline; ^#^*P* < 0.05, ^##^*P* < 0.01 vs. Saline + Cocaine (one-way ANOVA followed by Fisher’s PLSD test; *Chi* square test was done for convulsing and survival ratio).

**Fig. (3):**
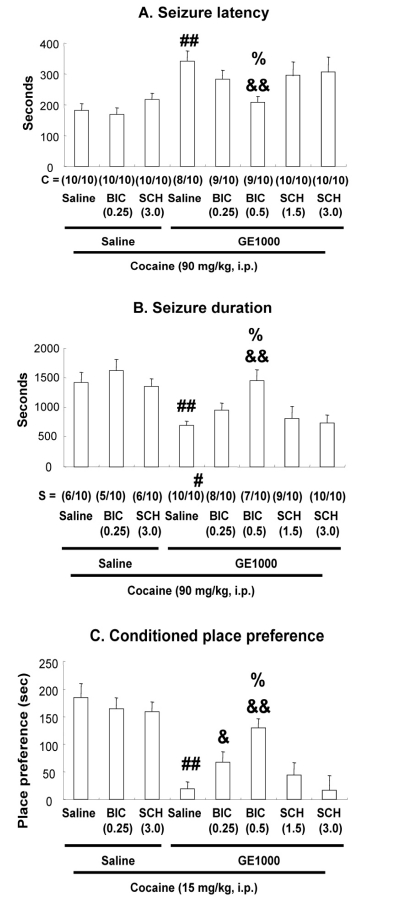
Effect of bicuculline or SCH 50911 on the GE-mediated pharmacological actions in response cocaine-induced seizure latency (**A**), seizure duration (**B**), and CPP (**C**). GE 1000 = GE 1000 mg/kg, BIC (0.25) or BIC (0.5) = bicuculline 0.25 or 0.5 mg/kg, SCH (1.5) or SCH (3.0) = SCH 50911 1.5 or 3.0 mg/kg, C = convulsing ratio, S = survival ratio. Each value is the mean ± S.E.M. of 10 (A and B) or 12 mice (C). ^#^*P* < 0.05, ^##^*P* < 0.01 vs. Saline + Saline + Cocaine; ^&^*P* < 0.05, ^&&^*P* < 0.01 vs. Saline + GE 1000 + Cocaine; ^%^*P* < 0.05 vs. BIC 0.25 + GE 1000 + Cocaine (one-way ANOVA followed by Fisher’s PLSD test; *Chi* square test was done for convulsing and survival ratio).

All mice receiving saline plus cocaine (90 mg/kg, i.p.) showed strong seizure behaviors with a latency of 188.3 ± 22.1 seconds. Treatment with GE significantly delayed cocaine-induced seizure onset, and significantly shortened seizure duration [for both of seizure latency and seizure duration: GE (500 mg/kg) + cocaine vs. Saline + cocaine, *P* < 0.05; GE (1000 mg/kg) + cocaine vs. Saline + cocaine, *P* < 0.01] in a dose-dependent manner. In addition, GE significantly increased survival rate [GE (1000 mg/kg) + cocaine vs. Saline + cocaine, *P* < 0.05] after cocaine-induced seizures, however GE did not affect significantly convulsing rate (Fig. **2A** and **2B**). Since GE has been reported to enhance GABAergic neurotransmission, we applied GABA_A_ receptor antagonist bicuculline and GABA_B_ receptor antagonist SCH 50911 to understand whether GABAergic receptors are involved in the anti-convulsive effect of GE against cocaine toxicity. Bicuculline (0.25 or 0.5 mg/kg, i.p.) or SCH 50911 (1.5 or 3.0 mg/kg, i.p.), at the doses we used here, did not induce any seizure behavior, or affect seizure activity. GE-mediated anticonvulsant effect was significantly reversed by bicuculline [for both of seizure latency and seizure duration: bicuculline (0.5 mg/kg) + GE (1000 mg/kg) + cocaine vs. saline + GE (1000 mg/kg) + cocaine, *P* < 0.01] in a dose-related manner, but not by SCH 50911, suggesting that GE attenuates cocaine-induced seizures, at least in part, via GABA_A_ receptors activation (Fig. **3A** and **3B**). Our results were consistent with previous report [7], which has shown that GABA_A_ receptor-positive modulator inhibits cocaine-induced seizures. In addition, another finding [24] suggested that cocaine suppresses the hippocampal inhibitory GABA_A_ current, and that this suppression may contribute to cocaine-induced seizures, which are in line with our results.

### GE Attenuated Cocaine-Induced CPP, but not Behavioral Sensitization

Although acute treatment with GE (1000 mg/kg, p.o.) significantly decreased locomotor activity (vs. Saline, *P* < 0.05, data not shown), mice received GE repeatedly did not show any significant difference in basal place preference (Fig. **2C**) or locomotor activity (Fig. **2D**) as compared with saline-treated mice. Cocaine (15 mg/kg, i.p.)-induced CPP was significantly blocked by GE [GE (500 mg/kg) or GE (1000 mg/kg) + cocaine vs. Saline + cocaine, P < 0.01] (Fig. **2C**), whereas GE failed to attenuate behavioral sensitization induced by cocaine (Fig. **2D**).

Similarly, Filip *et al*. [18, 21] showed that some GABA-mimetic agents, which increase synaptic GABA transmission, have showed different effects on cocaine-induced behavioral sensitization and self-administration, suggesting that unknown neuropharmacological mechanisms are also involved in the anti-cocaine effects of these GABA-mimetic agents. Since GE has been reported to modulate not only GABAergic system, but also other neurotransmitter systems [13, 16], it remains to be explored on this discrepancy using precise pharmacological tools/parameters. In addition, it has been demonstrated that the expression of cocaine sensitization requires GABAergic modulation, which showing decreased GABAergic responses [22] in the striatum and nucleus accumbens, while increased GABAergic responses in the medial prefrontal cortex [23].

In order to examine whether specific GABAergic receptors are involved in the GE-mediated pharmacological action in response to cocaine-induced CPP, GABA_A_ receptor antagonist bicuculline and GABA_B_ receptor antagonist SCH 50911 were used in the present study. Bicuculline (0.25 or 0.5 mg/kg, i.p.) or SCH 50911 (1.5 or 3.0 mg/kg, i.p.), at the doses we used, did not affect cocaine-elicited CPP. GE-mediated attenuation on cocaine-induced CPP was significantly reversed by bicuculline [bicuculline (0.25 mg/kg) or bicuculline (0.5 mg/kg) + GE (1000 mg/kg) + cocaine vs. saline + GE (1000 mg/kg) + cocaine, P < 0.05 or P < 0.01] in a dose-related manner, but not by SCH 50911, suggesting that stimulation of GABA_A_ receptors may be essential for the pharmacological action of GE (Fig. **3C**). Thus, our results may be in line with the previous finding, which has shown that GABA_A_ receptor positive-modulators inhibit rewarding properties of cocaine [24]. In addition, it has been reported that intra-hippocampal injection of muscimol, a GABA_A_ receptor agonist, inhibits context acquisition and expression of CPP paradigm after cocaine injection [25], suggesting that modulation of hippocampal GABAergic system is important for context-cocaine associations. Thus, it remains to be explored whether this area is involved in the GE-mediated attenuation of CPP induced by cocaine.

Taken together, our finding suggests that treatment with GE attenuates cocaine-induced seizures and CPP via, at least in part, GABA_A_ receptor activation.

## Figures and Tables

**Fig. (1):**
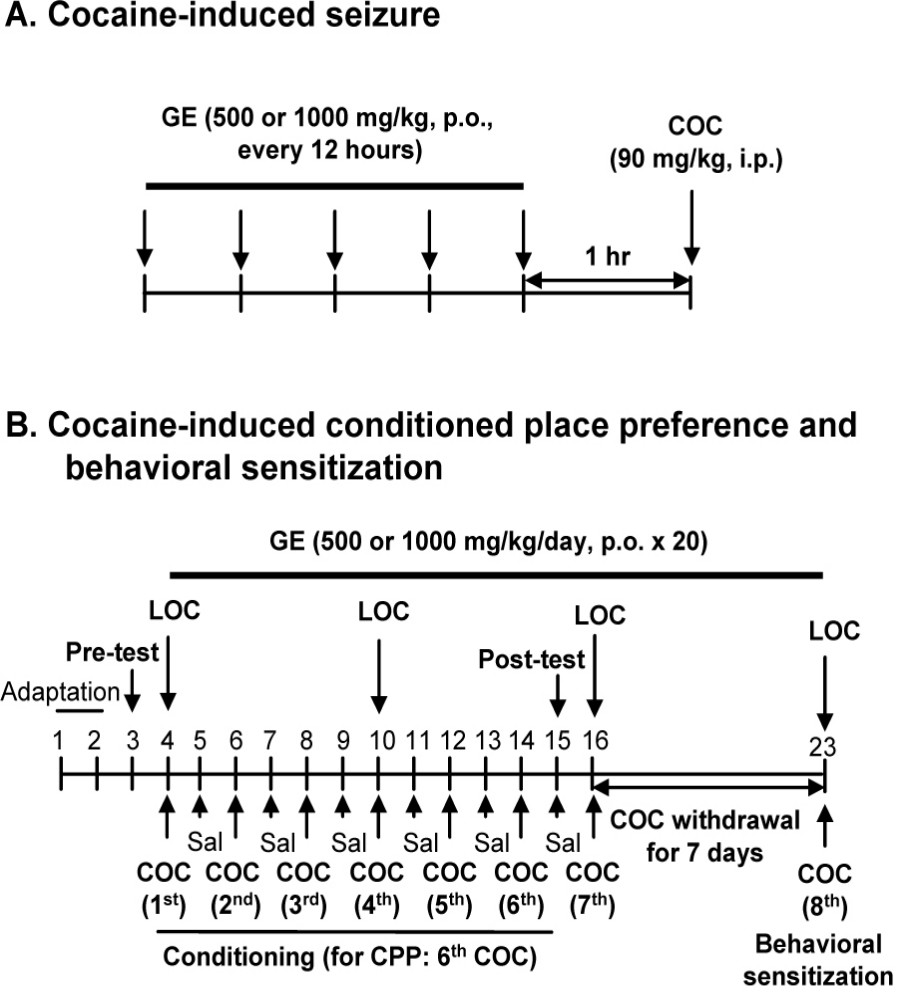
Experimental schedules for evaluating the effect of GE on the cocaine-induced seizures (**A**), CPP (**B**), and behavioral sensitization (**C**). COC = cocaine, LOC = locomotor activity. For CPP, mice received cocaine (15 mg/kg/day, i.p.) once every 2 days for 12 days. For behavioral sensitization, cocaine (15 mg/kg, i.p.) was administered after 7 days of withdrawal from cocaine. Bicuculline or SCH 50911 was administered 15 min before every cocaine injection.

## References

[1] United Nations Office on Drugs and Crime (2009). World Drug Report 2009.

[2] Koob GF (1998). Circuits, drugs, and drug addiction. Adv. Pharmacol.

[3] Lason W (2001). Neurochemical and pharmacological aspects of cocaine-induced seizures. Pol. J. Pharmacol.

[4] Anderson SM, Pierce RC (2005). Cocaine-induced alterations in dopamine receptor signaling: Implications for reinforcement and reinstatement. Pharmacol. Ther.

[5] Jayaram P, Steketee JD (2004). Effects of repeated cocaine on medial prefrontal cortical GABA_B_ receptor modulation of neurotransmis-sion in the mesocorticolimbic dopamine system. J. Neurochem.

[6] Li Y, Hu X-T, Berney TG, Vartanian AJ, Stine CD, Wolf ME, White FJ (1999). Both glutamate receptor antagonists and prefrontal cortex lesions prevent induction of cocaine sensitization and associated neuroadaptations. Synapse.

[7] Ushijima I, Kobayashi T, Suetsugi M, Watanabe K, Yamada M, Yamaguchi K (1998). Cocaine: evidence for NMDA-, ?-carboline- and dopaminergic-mediated seizures in mice. Brain Res.

[8] Ye JH, Ren J (2006). Cocaine inhibition of GABA_A_ current: role of dephosphorylation. Crit. Rev. Neurobiol.

[9] Hayashi J, Sekine T, Deguchi S, Lin Q, Horie S, Tsuchiya S, Yano S, Watanabe K, Ikegami F (2002). Phenolic compounds from Gastrodia rhizome and relaxant effects of related compounds on isolated smooth muscle preparation. Phytochemistry.

[10] Kim HJ, Moon KD, Oh SY, Kim SP, Lee SR (2001). Ether fraction of methanol extracts of Gastrodia elata, a traditional medicinal herb, protects against kainic acid-induced neuronal damage in the mouse hippocampus. Neurosci. Lett.

[11] Zeng X, Zhang S, Zhang L, Zhang K, Zheng X (2006). A study of the neuroprotective effect of the phenolic glucoside gastrodin during cerebral ischemia *in vivo* and *in vitro*. Planta Med.

[12] Jung JW, Yoon BH, Oh HR, Ahn JH, Kim SY, Park SY, Ryu JH (2006). Anxiolytic-like effects of Gastrodia elata and its phenolic constituents in mice. Biol. Pharm. Bull.

[13] Chen PJ, Hsieh CL, Su KP, Hou YC, Chiang HM, Sheen LY (2009). Rhizomes of Gastrodia elata BL possess antidepressant-like effect via monoamine modulation in subchronic animal model. Am. J. Chin. Med.

[14] Ha JH, Lee DU, Lee JT, Kim JS, Yong CS, Kim JA, Ha JS, Huh K (2000). 4-Hydroxybenzaldehyde from Gastrodia elata Bl. is active in the antioxidation and GABAergic neuromodulation of the rat brain. J. Ethnopharmacol.

[15] Ahn EK, Jeon HJ, Lim EJ, Jung HJ, Park EH (2007). Anti-inflammatory and anti-angiogenic activities of Gastrodia elata Blume. J. Ethonopharmacol.

[16] Shuchang H, Qiao N, Piye N, Mingwei H, Xiaoshu S, Feng S, Sheng W, Opler M (2008). Protective effects of gastrodia elata on aluminium-chloride-induced learning impairments and alterations of amino acid neurotransmitter release in adult rats. Restor. Neurol. Neurosci.

[17] Zeng X, Zhang Y, Zhang S, Zheng X (2007). A microdialysis study of effects of gastrodin on neurochemical changes in the ischemic/reperfused rat cerebral hippocampus. Biol. Pharm. Bull.

[18] Filip M, Frankowska M, Golda A, Zaniewska M, Vetulani J, Przegalinski E (2006). Various GABA-mimetic drugs differently affect cocaine-evoked hyperlocomotion and sensitization. Eur. J. Pharmacol.

[19] Gasior M, Ungard JT, Witkin JM (1999). Preclinical evaluation of newly approved and potential antiepileptic drugs against cocaine-induced seizures. J. Pharmacol. Exp. Ther.

[20] Shin EJ, Bing G, Chae JS, Kim TW, Bach JH, Park DH, Yamada K, Nabeshima T, Kim HC (2009). Role of microsomal epoxide hydrolase in methamphetamine-induced drug dependence in mice. J. Neurosci. Res.

[21] Filip M, Frankowska M, Zaniewska M, Golda A, Frzegalinski E, Vetulani J (2007). Diverse effects of GABA-mimetic drugs on cocaine-evoked self-administration and discriminative stimulus effects in rats. Psychopharmacology (Berl).

[22] Jung BJ, Dawson R, Sealey SA, Peris J (1999). Endogenous GABA release is reduced in the striatum of cocaine-sensitized rats. Synapse.

[23] Jayaram P, Steketee JD (2005). Effects of cocaine-induced behavioural sensitization on GABA transmission within rat medial prefrontal cortex. Eur. J. Neurosci.

[24] Barrett AC, Negus SS, Mello NL, Caine SB (2005). Effect of GABA agonists and GABA-A receptor modulators on cocaine- and food-maintained responding and cocaine discrimination in rats. J. Pharmacol. Exp. Ther.

[25] Meyers RA, Zavala AR, Speer CM, Neisewander JL (2006). Dorsal hippocampus inhibition disrupts acquisition and expression, but not consolidation, of cocaine conditioned place preference. Behav. Neurosci.

